# Influence of Smoking on Periodontal and Implant Therapy: A Narrative Review

**DOI:** 10.3390/ijerph20075368

**Published:** 2023-04-03

**Authors:** Marwa Madi, Steph Smith, Sami Alshehri, Osama Zakaria, Khalid Almas

**Affiliations:** 1 Department of Preventive Dental Sciences, College of Dentistry, Imam Abdulrahman Bin Faisal University, P.O. Box 1982, Dammam 31441, Saudi Arabia; 2 Department of Biomedical Dental Sciences, College of Dentistry, Imam Abdulrahman Bin Faisal University, P.O. Box 1982, Dammam 31441, Saudi Arabia

**Keywords:** cigarette smoking, periodontal disease, periodontal surgery, smoking cessation, peri-implantitis, sinus lifting, literature review

## Abstract

Background: smoking is considered the most modifiable risk factor for periodontal disease. Objective: the aim of this narrative review is to emphasize the effect of smoking on periodontal and implant therapy. Methods: The authors reviewed the literature reporting the clinical outcomes of smoking on periodontal surgical and nonsurgical treatment. The impact of smoking on implant therapy and sinus lifting procedures were also reviewed. Results: Periodontal and implant therapy outcomes are adversely affected by smoking. Smokers respond less favorably to periodontal therapy and periodontal flap procedures as compared to nonsmokers. Clinical outcomes for smokers are 50–75% worse than for nonsmokers. Studies reveal that smokers experience a significantly lower reduction in pocket depth compared to nonsmokers as well as less bone growth after treating infra-bony defects with guided tissue regeneration. The relative risk of implant failure is significantly higher in patients who smoke 20 cigarettes or more per day compared to nonsmokers. Additionally, smoking has also been shown to increase postoperative wound dehiscence and infection rates following sinus floor elevation. Longitudinal studies on smoke cessation have shown a reduction in bone loss and probing depths for periodontitis patients after cessation compared to those who smoke. Conclusion: Smoking cessation can reduce probing depths and improve clinical attachment after nonsurgical periodontal therapy. There is insufficient evidence regarding the effect of smoking on peri-implantitis, as well as the loss of implants in the long-term.

## 1. Introduction

The prediction of treatment outcomes is essential during periodontal and implant treatment planning. A growing focus has been placed on patient-related risk factors which can influence such treatment outcomes. These include systemic and environmental conditions, such as smoking [1]. After bacterial biofilm, tobacco smoking has been linked to periodontal disease as a major risk factor [2]. Smokers are more likely to present greater severity and extent of periodontitis as a result of smoking, according to evidence gathered from cross-sectional and cohort studies [3]. A causal association between smoking and tooth loss is also highly likely [4,5,6]. It is well known that smoking increases the risk of periodontitis-associated tooth loss and the loss of bone around dental implants [7]. Additionally, a growing number of patients who smoke or have smoked may require dental implants as a result of improved therapies and personalized medicine [8]. Using dental implants, a wide variety of prosthetic rehabilitation procedures can be performed [9]. It is important to note, however, that smoking increases the risk of peri-implant disease [10,11]. As long as the patient maintains proper oral health and ceases smoking, an implant-fixed prosthesis can have a longer survival rate and provide a better quality of life [12].

Controversy exists regarding smoking-related microbial diversity changes contributing to periodontitis [13]. The peri-implant microbiome has been shown to differ between smokers and nonsmokers [14]. The clinical significance of the effects of nonsurgical periodontal therapy, including adjunctive antimicrobial photodynamic therapy, remains doubtful [15,16,17]. This narrative review will present dental practitioners with an updated understanding of various aspects of the relationship between smoking, periodontal disease, tooth loss, periodontal and implant therapy, as well as smoking cessation. This will furthermore enable practitioners to make informed treatment decisions regarding smoker patients suffering from periodontal disease, as well as patients undergoing implant therapy.

## 2. Materials and Methods

### 2.1. Search Strategy

An electronic search was conducted in the following three databases: MEDLINE, Web of Science, and SCOPUS. A search was conducted in all databases from their earliest records up until May 2022. The searches were limited to publications in English. We manually searched the bibliographies of all relevant articles and review articles. Data that have not been published were not included. The literature search was conducted using the following keywords: cigarette smoking; periodontal disease; periodontal surgery; microbiome; tooth loss; antimicrobial photodynamic therapy; smoking cessation; smoking and bone regeneration; peri-implantitis; and sinus lifting.

### 2.2. Study Inclusion and Exclusion Criteria 

In this narrative review, we reviewed prospective and retrospective clinical studies that assessed the effects of smoking on periodontal tissues and microbiome following periodontal treatment and/or implant placement. Additionally, we included clinical trials comparing different interventions that reported results for smokers and nonsmokers separately. 

Our inclusion criteria included (1) publications written in English; (2) human studies; and (3) studies that classified subjects into two groups and included both smokers and nonsmokers.

We excluded studies that lacked sufficient data to allow a clear comparison between the regeneration of bone and tissue in smokers and nonsmokers after treatment.

## 3. Results

### 3.1. Smoking as a Risk Factor

Various researchers have demonstrated that smoking is correlated with a 40% prevalence of periodontal disease, with an odds ratio (OR) of 5.4 for periodontitis in smokers [18]. Smoking is associated with a two to six times increased risk of periodontal disease. In a study of 240 dental patients, Calsina et al. [19] found that smokers have a 2.7-fold greater likelihood of developing periodontal disease compared with nonsmokers. They also observed that former smokers have a 2.3-fold greater likelihood of periodontal disease development. According to Linden and Mullally [20], young smokers with periodontal disease have also been found to have an OR as high as 14.1. Hyman and Reid [21] examined the data from the National Health and Nutrition Examination Survey III and found a relative risk (OR) of 18.6 for ≥3 mm attachment loss among 20–49-year-old smokers. Loss of attachment ≤4 mm increased the OR to 25.6% among those over 50 years of age [21]. 

A recent publication by Bergström [22] noted that smoking as a risk factor is dependent upon the definition and prevalence of disease. The OR remained 3.0 with a broad definition of disease (1% pockets ≥5 mm). However, for a restricted definition of the disease (15% of pockets ≥5 mm), the OR ratio was 12.1. Thus, greater magnitudes of risk were correlated with increasing exposures. The combination of heavy exposure and a narrower disease definition has been shown to increase risk, with an OR ranging between 9.8 and 20.3 [22]. There is further support for the hypothesis that tobacco use is a periodontal disease risk factor based on the ability to report dose-response and duration of exposure to tobacco products [23,24,25,26,27]. Depending on the severity of periodontal disease and the amount of smoking, the risk of periodontitis has been indicated to increase by two to eight times, which includes bone loss and attachment loss [28]. 

### 3.2. Smoking and the Periodontal Microbiome

Smoking affects the human microbiome directly, or indirectly via immunosuppressive mechanisms, oxygen deprivation, or biofilm formation [29]. Tobacco smoke contains many chemicals that promote biofilm formation by increasing the adherence of bacteria to a stratum [30]. Subgingival microbial communities in smokers have been described to be less diverse than those of nonsmokers, including significant differences in the prevalence and abundance of disease-associated and health-compatible bacteria [31]. When exposed to tobacco, anaerobic conditions are induced that favor the early acquisition and quite unstable initial colonization of facultative anaerobic periodontopathogens in both marginal and subgingival oral biofilms [2]. As a result, marginal and subgingival biofilm communities can represent anaerobic microbiomes rich in pathogens and low in commensal bacteria over time [32,33,34]. A longitudinal study found that smokers’ subgingival bacteria are more diverse during and after naturally occurring gingivitis [2,35]. The detection rates of *Tannerella forsythia*, *P. gingivalis*, and *Prevotella intermedia* have been found to be higher in smokers than in nonsmokers in periodontitis patients [36]. Among the major determinants of a subgingival bacterial community shift induced by smoking, *Fusobacterium nucleatum* has been found to be more abundant in smokers than in nonsmokers [37,38]. 

Smokers with periodontitis have also been shown to have a robust core microbiome dominated by anaerobic bacteria [39,40]. 

There is no doubt that smoking negatively impacts subgingival microflora, but the underlying mechanisms remain unknown. When considering microbial diversity, different results have been reported in different studies [2,41]. Various indices and bioinformatic data processing methods to obtain sequences have been used in recent studies, leading to different conclusions [42]. Thus, there is no consensus on how smoking-related changes in microbial diversity contribute to periodontitis as of yet [2]. 

Smoking has been suggested to potentially cause a shift in the essential metabolic functions of the oral microbiome, thereby inducing variation in the composition of the whole microbiome between smokers and nonsmokers. This includes a depletion of bacterial genera related to carbohydrate, energy, and xenobiotic metabolism [43]. There is evidence that cigarette smoke extract (CSE) exposure regulates the DNA repair genes as well as the virulence genes of *P. gingivalis* [44,45,46]. Nicotine and *P. gingivalis* lipopolysaccharide (LPS) treatment of dendritic cells (DCs) has been shown to modulate the immunopathogenesis of periodontal diseases, including the modulation of LPS-stimulated DC immunoregulatory functions [32,47]. Smokers have more severe periodontal disease than nonsmokers, and it has been speculated that nicotine combined with *P. gingivalis* or *P. gingivalis* LPS causes collagen degradation and bone resorption by tipping the balance between matrix metalloproteinases (MMPs) and tissue inhibitors of metalloproteinases (TIMPs) [2,48,49].

There have been a few studies investigating the ability of periodontal pathogens to colonize epithelial cells following exposure to harmful substances from smoking, but the results have been conflicting [2,13,50]. Low concentrations of cigarette smoke condensate have been shown to increase the invasion of human gingival epithelial cells by *P. gingivalis* [46]. Studies combining the treatment of gingival epithelial cells with low concentrations of CSE and *P. gingivalis* showed the inhibition of wound closure, and *P. gingivalis* invasion was shown to increase near the wound area [32,50]. Summary of the research findings are presented in Table 1. However, due to variations in study designs, no correlation between periodontal pathogen colonization and smoking was observed [2,13].

### 3.3. Smoking and Tooth Loss

Various reports on the relationship between smoking and periodontitis progression have described a negative association between smoking and the number of teeth, with smokers being reported to have fewer teeth [3,6,51]. Systematic reviews have shown strong evidence for a dose-response relationship between smoking and tooth loss [4]. Meta-analyses of cross-sectional studies have found no differences regarding risk between former and current smokers, regardless of whether patients are edentulous, have lost one or more teeth, or have lost eight or more teeth [3].

A large cohort study which included 23,376 individuals with a mean age of 50 y (range 20 to 70 y) was performed in a German population [5]. Smoking exposure among participants was determined by utilizing exclusive categories: never smokers, former smokers (<10, 10 to <20, ≥20 years since cessation), and current smokers (<15 and ≥15 cigarettes/day). Smoking was associated with tooth loss more frequently in males than in females and among younger people than in older ones. There was a 3 times greater risk of tooth loss in males who smoked (15 cigarettes a day; 95% confidence interval, 3.0, 4.4) and over twice the risk of tooth loss in smokers‘ female younger than 50 years of age (odds ratio, 2.5; 95% confidence interval, 2.1, 2.9) when compared to nonsmokers. 

A cross-sectional study in Finland was performed on 5,540 subjects with good oral health and who had access to subsidized dental care since childhood [6]. In this study, education, tooth brushing frequency, alcohol use, and diabetes were identified as potential confounders. Current smokers were those who reported smoking at least occasionally. Former smokers included those who had smoked daily for at least one year but had stopped smoking and were not smokers at the time of the study. Never smokers included all participants who had smoked daily for less than one year in their lifetime and were not smokers at the time of follow-up [6]. Subjects reported the number of teeth they had at the age of 46 with no distinction for third molars from other teeth, and the number of teeth varied with smoking status: the percentages of those with fewer than 28 teeth among current smokers, former smokers, and never smokers was found to be 49%, 42% and 35%, respectively. 

Second-hand smoke (SHS), in which people breathe in smoke exhaled by smokers, is also considered as a risk factor for oral diseases, including the increased risk for periodontal disease [51,52,53]. A study in Japan [54] evaluated the association between SHS experience and the number of remaining teeth among nonsmoking Japanese individuals, specifically in an older population aged ≥65 years. The data of 18,865 respondents who had never smoked were analyzed. The study found that daily SHS was related with fewer remaining teeth, with an odds ratio for having no teeth rather than having more than 20 teeth of 1.35 (*p* < 0.01) [54]. 

### 3.4. Periodontal Treatment in Smokers

Researchers have conducted numerous clinical studies on the responses of smokers and nonsmokers to various types of periodontal therapy, including nonsurgical as well as surgical procedures [20]. Smoking has been demonstrated to negatively impact all types of periodontal therapy, as up to 90% of patients with refractory periodontitis have been shown to be smokers [21]. Researchers have found that nonsmokers have significantly reduced probing depths, less bleeding, and improved clinical attachment after nonsurgical and surgical treatments [22,23]. A similar finding has been observed after regenerative procedures [24] and when treating furcation lesions [25]. 

### 3.5. Nonsurgical Therapy

Studies have shown that current smokers show less significant improvements in clinical responses, such as reduction in PPD and gain in CAL, after SRP than nonsmokers or past smokers [16,55]. However, Jin et al. [55] have found significantly greater reductions in the order of 1.0 mm in nonsmokers than in smokers at 1 and 3 months following nonsurgical therapy. Furthermore, a study by Poucher et al. [56] reported that both nonsmokers and smokers experienced the same level of relief from nonsurgical therapy after nine months, based on a reduction in probing depth, an increase in clinical attachment levels, and fewer bleeding episodes after periodontal probing. The gingival index, however, only improved by a significant amount after 9 months in comparison to the gingival index in nonsmokers. In Zuabi et al.’s study [57], they selected 12 smokers and 14 nonsmokers and studied their post-treatment probing depths and clinical attachment levels. They concluded that there was no significant difference between smokers and nonsmokers. In their study, smokers exhibited significantly more plaque accumulation than nonsmokers. In addition to their deeper probing depths at baseline, smokers also had significantly greater probing depths than nonsmokers. Therefore, the lower pocket reduction in smokers compared to nonsmokers could be attributed to greater pocket depths in smokers prior to treatment.

Additionally, supragingival periodontal treatment has been shown to only slightly affect the diversity of subgingival microbiota in smokers compared with nonsmokers [15], and this may account for smokers responding less favorably to nonsurgical periodontal treatment than nonsmokers [58].

Meinberg et al. [59] reported significantly greater bone loss in smokers in comparison to nonsmokers after a 12-month follow-up period and concluded that more long-term studies are required in order to resolve the association between smoking status and outcome variables. Despite the fact that most clinical trials have been conducted in patients with periodontitis, Darby et al. [60] showed a significant reduction in probing depths for nonsmokers (2.4 mm), compared with smokers (1.3 mm) suffering from previously diagnosed aggressive periodontitis. After scaling and root debridement, an inferior reduction in probing depths following therapy for smokers was observed in both aggressive and chronic periodontitis, revealing the systemic effects of smoking on the host response and the healing process.

### 3.6. Nonsurgical Therapy in Combination with Local and Systemic Drug Delivery

Preshaw et al. [51] found that adjunctive use of subantimicrobial-dose doxycycline (Periostat) therapy improved therapeutic outcomes for smokers and nonsmokers treated by scaling and root planing. Nicotine-dependent smokers on doxycycline therapy were shown to achieve almost the same level of clinical improvement (probing depth reduction and clinical attachment gain) as nonsmokers on placebo therapy. Williams et al. [61] found that minocycline microspheres were able to lower the levels of plaque and calculus as an adjunct therapy to scaling and root planing, resulting in the additional benefit of 0.3 mm in clinical attachment gain. By increasing the percentage of treatment sites experiencing probing depth reductions of at least 2 mm, a significant increase in the response to treatment was described. 

Another review study [62] of smoker patients treated with nonsurgical periodontal therapy or supportive periodontal therapy together with systemic adjunctive host modulation therapy, which included the use of low-dose doxycycline and low-dose flurbiprofen, demonstrated improvement in clinical parameters. These differences were more pronounced in moderate (4 to 6 mm) and deep (≥7 mm) periodontal pockets. Smokers receiving adjunctive host modulation therapy also demonstrated a decrease in the proportion of sites with red-complex putative periodontal pathogens. Furthermore, a significantly greater reduction in gingival crevicular fluid levels of proinflammatory cytokines was seen in patients receiving scaling and root planing and systemic adjunctive host modulation therapy than in those receiving scaling and root planing alone [62]. 

### 3.7. Smoking and Antimicrobial Photodynamic Therapy

Antimicrobial photodynamic therapy (aPDT) utilizes photosensitizing agents with different sources of light, such as lasers or light-emitting diodes (LEDs), for the purposes of promoting the generation of reactive oxygen species such as free radicals and singlet oxygen, which are cytotoxic to certain bacteria [63]. A randomized controlled clinical trial utilizing indocyanine green (ICG)-mediated antimicrobial photodynamic therapy (aPDT) as an adjunct to scaling and root planing was conducted on 29 patients, including three months of follow-up. ICG is a photosensitizer with better tissue absorption and low toxicity. A significant reduction in probing depths and clinical attachment gain was observed in the ICG group as compared to the scaling and root planing treatment alone [64]. Over 3 months, a double-blind, randomized, controlled clinical trial found that multiple sessions of aPDT as an adjunct to surgical periodontal treatment significantly improved clinical parameters at 90 days postoperation [65]. A systematic review and meta-analysis demonstrated a more pronounced effectiveness of aPDT when combined with scaling and root planing, rather than aPDT monotherapy [66].

A randomized, prospective, controlled clinical trial was conducted with a follow-up period of 180 days to determine the effects of conventional periodontal therapy (scaling and root planing) in combination with either metronidazole (MTZ) plus amoxicillin (AMX) or multiple applications of aPDT in patients who smoke [64]. Microbiological analysis was performed in this study, and there was a reduction in the levels of *P. gingivalis*, *P. intermedia,* and *P. nigrescens* at 180 days in the MTZ + AMX group, compared to the baseline, whereas the aPDT group showed a reduction in the levels of *P. intermedia* and *P. nigrescens* at 180 days and in *P. nigrescens* at 90 days. In contrast, scaling and root planing treatment did not cause a significant reduction in the levels of microorganisms compared to the baseline. The study concluded that the adjunctive use of MTZ + AMX or three sessions of aPDT constituted an effective therapy for the treatment of periodontitis in smokers [63].

Another randomized clinical trial was performed to assess the efficacy of scaling and root planing (SRP) with and without adjunct aPDT in the treatment of chronic periodontitis among smokers and never smokers [67]. This study found no statistically significant differences at 1-month and 3-months follow-ups in periodontal parameters among smoker individuals that received either SRP alone or SRP with adjunct aPDT, as well as no statistically significant difference in periodontal parameters among nonsmoker individuals that received SRP alone and SRP with adjunct aPDT. The authors concluded that the outcomes of SRP with or without aPDT for the treatment of periodontitis are compromised in cigarette smokers as compared to non-smokers. Among never smokers with periodontitis, the outcomes of SRP with or without aPDT were found to be comparable. Thus, the significance of aPDT was found to be questionable in reducing periodontal inflammation. However, aPDT was only performed once at baseline in this study, and the authors did suggest that additional sessions of SRP with adjunct aPDT (for example at 1-month follow-up) could have resulted in significant differences in periodontal inflammatory parameters among individuals that received SRP alone and SRP with adjunct aPDT at 3-months follow-up [67]. In contrast, a randomized clinical trial showed that multiple episodes of aPDT adjunctive to nonsurgical treatment in smoker patients with chronic periodontitis did not significantly improve the clinical, immunological, and microbiological parameters when compared with SRP alone [68]. A systematic review on the clinical efficacy of aPDT adjunctive to scaling and root planing in the treatment of chronic periodontitis showed no significant clinical improvements in probing depths and clinical attachment loss in smoker patients. Furthermore, the results of subgroup analysis revealed a negative impact of smoking on the clinical efficacy of combined therapy using both SRP and aPDT [17]. 

### 3.8. Surgical Treatment

Clinical studies have shown diminished outcomes of the healing response following periodontal surgery in smokers compared with nonsmokers [69,70]. Trombelli et al. [69] randomized, controlled clinical trial reported the treatment outcome in periodontal furcation defects following periodontal flap surgery compared between cigarette smokers and nonsmokers. Six-month follow-up results revealed twice as much gain in the clinical attachment level (CAL) in nonsmokers than in smokers.

Other studies have also supported a trend for less favorable healing following periodontal surgical procedures in smokers as well as an increased risk of relapse during post-surgical maintenance [71,72]. 

Clinical results of studies have shown significantly less reduction in probing depth among smokers as compared to nonsmokers. A reduced probing depth has been found in smokers as well as in nonsmokers ranging from 0.7641 to 2.05 mm and from 1.2741 to 2.40 mm, respectively [73]. With regard to clinical attachment level (CAL), nonsmokers have been shown to gain between 0.2939 and 1.6 mm compared to smokers who gained between 0.0939 and 1.2 mm [71].

The finding of a study by Kotsakis et al. [74] is in agreement with Johnson and Guthmiller [73], who reviewed the literature about periodontal therapy in smokers and concluded that smokers are eligible for periodontal surgery. They also suggested that periodontal surgery may be recommended for smokers in order to reduce pocket depth, although the clinical outcomes are expected to range from half to seventy per cent of those observed in nonsmokers. Hellström et al. [72] reported that the addition of a local antimicrobial in conjunction with a modified Widman flap procedure increased the reduction in mean probing depth by 0.3 mm when compared to using a modified Widman flap procedure alone, resulting in a significant number of pockets experiencing probing depth reductions of >2 mm. 

### 3.9. Effect of Smoking on Bone Regeneration

A study by Tonetti et al. [75] reported a larger clinical attachment gain of 5.2 mm for nonsmokers compared with smokers (2.1 mm) following guided tissue regeneration of infrabony defects with Gore-Tex membranes (Gore Medical Products, Newark, DE, USA). This study also included a one-year follow-up period. Furthermore, the authors concluded that the consistently higher plaque levels observed in smokers in comparison to nonsmokers will also influence clinical outcomes.

Studies have found that smoking has a significant effect on bone gain or bone fill after periodontal treatment, including studies conducted by Ehmke et al. [76], Heden et al. [77], Loos et al. [78], and Yilmaz et al. [79]. Following the use of a bioabsorbable membrane in intrabony defects, Ehmke et al. [76] reported a mean bone growth of 0.2 mm in smokers in contrast to 2.2 mm in nonsmokers. According to Heden et al. [77], smokers who underwent EMD (Enamel Matrix Derivative) therapy in intrabony defects experienced a bone loss of 2.6 mm compared to nonsmokers who underwent EMD therapy and showed a bone gain of 3.3 mm.

Two other studies [80,81] did not find a significant difference in bone gain between smokers and nonsmokers following treatment. Using EMD to treat intrabony defects was the subject of one study [81]. In the other study [80], no statistical analysis was performed to compare the results of smokers and nonsmokers within each of the test and control groups. Nevertheless, they did include a statistical analysis of the test group, which included 0.3 mg/mL of recombinant human platelet-derived growth factor (rhPDGF) and beta-tricalcium phosphate (b-TCP). A nonsignificant difference in bone density was found between smokers and nonsmokers [80]. According to Nevins et al. [80] and Trombelli et al. [81], there was no significant difference in bone resorption in smokers and nonsmokers following treatment with EMD [81] or b-TCP and rhPDGF [81]. 

### 3.10. Smoking Cessation and Periodontal Tissues

Domagala-Kulawik [82] reported that many changes in the immune system may still be present following smoking cessation (caused by tobacco). In contrast, Bouloukaki et al. [83] has suggested that within the first 6 months following smoking cessation, CD8+ T-cells increase and CD4+/CD8+ cells decrease. 

A limited number of systematic reviews have addressed the effects of smoking cessation on periodontal health [84]. Two systematic reviews with meta-analyses utilizing data from interventional studies assessed the influence of tobacco smoking cessation on the clinical outcomes of nonsurgical periodontal treatment [85,86]. Both reviews have reported that smoking cessation yields benefits in regard to probing depth and clinical attachment level in individuals who have received nonsurgical periodontal treatment. The systematic review by Leite et al. [87] showed that the risk for periodontitis incidence and progression could be reversed after smoking cessation to the same level as that of never smokers. The large cohort study by Dietrich et al. indicated that smoking cessation was consistently associated with a reduction in tooth loss risk. Furthermore, smoking cessation appears to reduce the excess risk of tooth loss in a time-dependent manner, with pronounced benefits evident within 10 years of quitting [5]. Similarly, a study in Finland found that 10–11 years of abstinence from smoking among men could result in a greater decrease in their risk for tooth loss [6]. A study by Ramseier et al. [88] has also stated that interventions for smoking cessation are effective, thus emphasizing the need for behavioral support in periodontal care. 

### 3.11. Smoking and the Peri-Implant Microbiome

Smoking has been reported to shape the peri-implant microbiome even in states of clinical health, which includes the depletion of commensals from healthy sites and enrichment for pathogens in the subgingival peri-implant microbiota [89,90]. However, a prospective cross-sectional study of patients with healthy implants found that although smokers presented with peri-implant microbiota composed of a greater number of periodontal pathogens than in nonsmoking patients, these differences were found to be not statistically significant [14]. A case-control study on the effects of smoking on the peri-implant microbiome in non-periodontitis subjects has described a core microbiome which is shared by at least 75% of individuals in the healthy peri-implant sulcus in smokers and nonsmokers, with nonsmokers presenting a core harbored by usually healthy phylotypes and some commensal species, whereas smokers presented a disease-associated core microbiome [91]. A study using 16S ribosomal RNA sequencing reported a lower bacterial diversity in the microbial signatures of the healthy peri-implant microbiome in smokers, with a significant enrichment for disease-associated species as compared to nonsmokers [92]. Furthermore, in this study, shifts from health to peri-implant mucositis in smokers were furthermore accompanied by the loss of several health-associated species, leading to a further decrease in diversity, and peri-implantitis did not differ significantly from mucositis in species richness, thereby suggesting that the pathogen-rich state established in mucositis persists in peri-implantitis in smoker patients. The study also showed that, in contrast, the shift from health to mucositis in nonsmoker patients resembled primary ecological succession which comprised the acquisition of several species without replacement of pioneer organisms, thereby creating a significant increase in diversity. Few differences were also detected between peri-implantitis and mucositis in nonsmokers. The study thus concluded that the transition from health to mucositis and progression to peri-implantitis takes an alternate pathway in smokers, which includes the further enrichment of the microbiome and a decrease in diversity [92]. Another cross-sectional study compared the peri-implant microbiota in smokers and nonsmokers and also demonstrated a significantly higher microbial richness in smoker patients around implants affected by peri-implantitis as compared to either healthy implants or implants presenting with mucositis [93]. 

### 3.12. Smoking and Dental Implants 

Shenava et al. [94] found that smokers had a higher implant failure rate (63% to 66%) compared to nonsmokers (36% to 37%) and concluded that smoking was not contraindicated, but that patients should be advised of its adverse effects. A systematic review conducted by Takamiya et al. [95] reached similar conclusions. Bain and Moy [96] studied the relationship between implant success and smoking, and they found that smokers experienced an implant failure rate of 11.28% compared to 4.76% for nonsmokers.

Shenava et al. [94] reported a survival rate of 30.95% for implants in patients with >10 years of smoking versus 69.05% for those with <10 years of smoking. In addition, they found a higher incidence of implant failure with cigarette consumption of >20 packets/year compared to a consumption of <20 packets/year, a difference which was not found to be statistically significant.

A study conducted by Twito and Sade [97] found that smokers had a higher failure rate for implants (5,6%) compared to nonsmokers (3,5%). According to Naseri et al. [98] in their systematic review and meta-analysis of the association between quantity of smoking and dental implant failure, smoking more than 20 cigarettes daily was associated with an increased relative risk of implant failure compared to nonsmokers.

### 3.13. Smoking and Peri-Implantitis

A systematic review by Heitz-Mayfield and Huynh-Ba [99] demonstrated an increased risk of peri-implantitis among smokers. In a follow-up study of 10 years, DeLuca et al. [100] reported a significantly higher failure rate among smokers compared to nonsmokers. The prevalence of peri-implantitis varies widely in the literature, ranging from 6.47 to 28% [101], depending upon the peri-implantitis definition, the follow-up period, and implant variables. There is particular evidence that smoking is a risk factor for peri-implantitis [102,103], with some studies showing that smoking has adverse effects on the treatment outcomes of peri-implantitis [104,105]. 

A study by Roos-Jansåker et al. [106] showed that after estimating the probability of peri-implantitis, smoker patients (303 implants) were more likely to develop peri-implantitis (univariate analysis: OR: 7.7, [98% CI: 2.5–14, *p* < 0.001); multivariate analysis: OR: 4.6 [98% CI: 1.1–19]) than nonsmokers (OR: 1.0 for both). The results of an implant-based meta-analysis by Sgolastra et al. [107] revealed that smokers were at a higher risk of peri-implantitis (RR: 2.17, 95% CI: 0.78–1.75, *p* = 0.46) than nonsmokers but not in a patient-based meta-analysis (RR: 1.17, 95% CI: 0.78–1.75, *p* = 0.46). Koldsland et al. [108] observed that implant loss was associated with the history of smoking. Table 2 summarizes studies reporting the influence of smoking on periimplantitis.

### 3.14. Smoking and Sinus Floor Elevation

Comparing smokers with nonsmokers, Peleg et al. [109] observed no significant differences in the long-term success rates of implants placed simultaneously with sinus grafting. Ghasemi et al. [110] observed that smoking increased the risk of postoperative wound dehiscence (WD) and infection; more precisely, smokers experienced a 7.8 times higher likelihood of WD after sinus floor elevation (SFE) than nonsmokers, and smokers experienced a 5.3 times higher likelihood of infection after SFE than nonsmokers. A study conducted by Chambrone et al. [111] examined the effects of smoking on the survival rate of implants placed in SFE areas. In the overall meta-analysis, smoking appeared to be associated with implant failure; however, this negative effect was not confirmed when only prospective studies were evaluated. In addition, flap tension and the use of barrier membranes over lateral windows may contribute to the development of incision line openings and WD [112]. Schwarz et al. [113] demonstrated that all cases of WD occurred at sites with prolonged incisions of more than a two-tooth gap.

## 4. Discussion

Studies support the hypothesis that tobacco use is a risk factor for periodontal disease [18,19,20,21,22,23,24,25,26,27]. Evidence suggests that as smoking increases, periodontal disease becomes more severe, and studies support the hypothesis that tobacco use may be considered a periodontal disease risk factor based on the ability to report dose-response and duration of exposure to tobacco products [19,20,21]. Greater magnitudes of risk of periodontal disease have been correlated with increasing smoking exposure [22]. However, it remains a challenge to estimate the magnitude of smoking as a risk factor, since measurement of a subject’s exposure to tobacco poses inherent problems [25,26,27]. Treatment for periodontal disease has been suggested to be likely more effective in nonsmokers than in smokers, with the response of former smokers being intermediate compared with smokers [19]. There is no doubt that smoking negatively impacts the subgingival microflora, but the underlying mechanisms remain unknown [2,41]. Tobacco exposure induces anaerobic conditions that affect the human microbiome directly, or indirectly via immunosuppressive mechanisms and oxygen deprivation, in both marginal and subgingival biofilm formation [2,29,30], which becomes rich in pathogens and low in commensal bacteria [32,33,34]. Smoking can potentially cause a shift in the essential metabolic functions of the oral microbiome, thereby inducing variation in the composition of the whole microbiome between smokers and nonsmokers. Periodontal pathogens are not directly affected by smoking, but virulence factors of bacteria can be altered by smoking [2,13,43]. *F. nucleatum*, which is more abundant in smokers than in nonsmokers, plays a critical role in the subgingival biofilm due to its “bridging species” role among microorganisms as well as its local immunosuppressive capability and, consequently, may thus facilitate periodontitis severity and progression [2,37,38]. Smokers with periodontitis have a robust core microbiome dominated by anaerobic bacteria [39]. Smoking also leads to a decrease in the ability of a subgingival microbiome to sustain its original state under distress following episodes of disease, thereby lowering the resilience of the ecosystem and decreasing its resistance to future disease [46].

A positive dose- and time-dependent correlation has been shown to exist between cigarette smoking and the risk of tooth loss [5]. Among middle-aged Finnish adults, even with lifelong access to subsidized dental care, current and former long-term smoking was shown to be associated with tooth loss, with a stronger association between smoking and tooth loss among men than among women [6]. 

Although there may be no significant differences between smokers and nonsmokers who undergo nonsurgical therapy, periodontal treatment in smokers tends to have less favorable therapeutic responses to nonsurgical therapy compared to nonsmokers [55]. Supragingival periodontal treatment may only slightly affect the diversity of subgingival microbiota [15], and greater pocket depths in smokers prior to treatment can result in less pocket reduction in smokers compared to nonsmokers. This may account for smokers responding less favorably to nonsurgical periodontal treatment than nonsmokers [15,57,58]. Clinical parameters may improve in both smoking and nonsmoking periodontitis patients following nonsurgical treatments; however, a lower reduction and greater post-therapy prevalence of periodontal pathogens may be observed in smokers [15,114], as a pathogenic subgingival biofilm is more likely to be re-established in smokers after treatment compared to nonsmokers [3,16]. Inferior reduction in probing depths following therapy for smokers in aggressive periodontitis may reveal the systemic effects of smoking on the host response and the healing process [60]. 

Studies have reported on the clinical outcomes in both smokers and nonsmokers following nonsurgical treatment with, or without, either systemic or local antimicrobial therapy [61,115]. The adjunctive use of systemic metronidazole and amoxicillin in scaling and root planing for patients suffering from periodontitis can lead to a reduction in the proportions and mean counts of periodontal pathogens in the subgingival microbial profile as well as an increase in the proportions of host-compatible species [116]. Emerging evidence has shown that both locally and systemically applied antimicrobials in smoker patients may enhance the outcomes of scaling and root planing procedures, including guided tissue regeneration [61,117]. The combination of scaling and root planing with adjunctive local therapy may represent a more effective way of treating smokers with periodontitis than mechanical therapy alone. Even if the degree of improvement experienced by smokers tends to be less than by nonsmokers, smokers who undergo combination therapy can benefit both in terms of probing depth and attachment level outcomes [118,119].

Antimicrobial photodynamic therapy (aPDT) utilizes photosensitizing agents with different sources of light, such as lasers or light-emitting diodes (LEDs), for the purpose of promoting the generation of reactive oxygen species such as free radicals and singlet oxygen, which are cytotoxic to certain bacteria [64]. It has been suggested that in patients who present a systemic modifying factor, such as smoking, aPDT could be beneficial by means of altering the periodontal tissue’s biological response, which includes the progression of periodontal disease as well as during tissue repair following conventional periodontal treatment [64]. However, outcomes of SRP with or without aPDT for the treatment of periodontitis are compromised in cigarette smokers as compared to never smokers, as shown in human randomized controlled clinical trials. Therefore, the efficacy of aPDT, when utilized in the nonsurgical management of periodontitis so as to improve treatment outcomes, remains debatable [67,68]. 

Diminished outcomes of the healing response may occur following periodontal surgery, including significantly less reduction in probing depths as well as an increased risk of relapse during post-surgical maintenance in smokers compared with nonsmokers [70,71,72,73,74,75,120,121] (Table 3). Smokers may experience a mean gain in post-surgical CAL; however, greater post-surgical CAL gain can be achieved in nonsmokers compared with smokers [72,74]. Periodontal flap procedures performed in smokers result in moderate improvements in clinical measurements of periodontal disease; however, the magnitude of the improvement may be considerably less than that experienced by nonsmokers [70,75,122,123]. 

Consistently higher plaque levels observed in smokers in comparison to nonsmokers can influence clinical outcomes [76]. Smoking has significant effects on bone gain or bone fill and following the usage of bioabsorbable membranes in intrabony defects, the mean bone growth in smokers may be far less than in nonsmokers [56,77,78,79,80]. Mixed results may be experienced regarding the significance of EMD therapy of intrabony defects, including smokers experiencing bone loss in contrast to the bone gain observed in nonsmokers [78,81,82]. Regenerative procedures utilizing b-TCP and rhPDGF may be able to overcome the negative effects of smoking [81,82]. 

Changes in the immune system following smoking cessation may occur within 6 months or longer. Smoking cessation has a positive effect on periodontal parameters in nonsurgical periodontal treatment, including probing depth, clinical attachment level, radiographical bone loss, and the risk of periodontitis and tooth loss [86,87,88]. Behavioral support for smoking cessation interventions is effective, including in the reduction of risk of tooth loss [5,6,88]. However, more prospective long-term studies on the impact of combined periodontal treatment with tobacco cessation programs are needed [85].

Smoking can shape the peri-implant microbiome even in states of clinical health, which includes the depletion of commensals from healthy sites and the enrichment for pathogens in the subgingival peri-implant microbiota [89,90]. In non-periodontitis subjects, 75% of individuals can share a core microbiome in the healthy peri-implant sulcus in smokers and nonsmokers, with smokers presenting with a more disease-associated core microbiome including a lower bacterial diversity [14,91]. The transition from health to peri-implant mucositis and progression to peri-implantitis in smokers includes further enrichment of the microbiome and a decrease in bacterial diversity [92,93]. In contrast, the shift from health to peri-implant mucositis in nonsmoker patients comprises a significant increase in bacterial diversity [92]. 

Controversy exists regarding significant differences in the incidence of peri-implantitis and the loss of implants between smokers and nonsmokers [105]. Although smoker patients may be more likely to develop peri-implantitis and experience higher implant failure rates as well as experience adverse effects on treatment outcomes of peri-implantitis [104,106,107], insufficient evidence supports smoking to be a risk factor for peri-implantitis [108].

Smoking may not be associated with late implant loss. The risk of postoperative wound dehiscence and infection in sinus lifting procedures is increased in smokers compared to nonsmokers and can be associated with implant failure [111,112,113].

Studies have identified smoking as being associated with early implant loss [123,124]. A 2- to 3-fold increase in early implant loss, although not statistically significant, has been reported among smokers compared with nonsmokers [125,126]. However, other studies have failed to identify smoking as a predictor for early implant loss [127,128].

A majority of studies have not identified smoking to be associated with late implant loss [129,130]. A history of smoking has been shown to be associated with late implant loss, and in contrast to smoking history, smoking status at the time of implant installation may not be a significant predictor of the outcome [131]. On the other hand, another study found that smoking at the time of implant surgery may be associated with late implant loss, as reported over an observation period of 4–16 years [132].

In this narrative review, we summarize clinical findings concerning the effect of smoking on periodontal and peri-implant tissues. This review has, however, the limitation of not being systematic. Additionally, the included studies were not categorized by smoker classifications or daily cigarette smoking patterns.

## 5. Conclusions

Smoking can impair the outcome of periodontal surgery in smokers compared to nonsmokers. Treatment outcomes of sinus floor elevation procedures are less predictable among smokers. Cessation of smoking has the potential to serve as an additional factor in facilitating the improvement of clinical outcomes of surgical and nonsurgical periodontal therapy, as well as implant therapy.

## Figures and Tables

**Table 1:** Smoking and the periodontal microbiome.

Studies	Findings
Jiang et al. 2020. [2] Shchipkova et al. 2010. [31] Joshi et al. 2014. [35] Mikhailova et al. 2017. [36] Moon et al. 2015. [37] Bizzarro et al. 2013. [41] Nearing et al. 2018. [42]	Bacterial diversity Different results have been reported in various studies regarding microbial diversity. Significant differences have been found in the prevalence and abundance of disease-associated and health-compatible bacteria. Smokers’ subgingival bacteria are more diverse during and after naturally occurring gingivitis and subgingival microbial communities may be less diverse than those of nonsmokers. *Fusobacterium nucleatum* has been found to be more abundant in smokers than in nonsmokers, and detection rates of *Tannerella forsythia, P. gingivalis*, and *Prevotella intermedia* have been found to be higher in smokers than in nonsmokers in periodontitis patients.
Jiang et al. 2020. [2] Hanioka et al. 2019. [32] Shapiro et al. 2022. [34] Bagaitkar et al. 2009. [44] Bagaitkar et al. 2011. [45] Bagaitkar et al. 2010. [46] Yanagita et al. 2012. [47] Zhang et al. 2010. [48] Kim et al. 2012. [49]	Bacterial virulence Smoking-induced alterations of microbial functions include the increase of virulence genes in pathogenic bacteria and reduces the host’s response to periodontal pathogens. Cigarette smoke extract (CSE) exposure regulates the DNA repair genes as well as the virulence genes of *P. gingivalis.* This can induce changes in the *P. gingivalis* phenotype which enables *P. gingivalis* to subsequently neutralize the proinflammatory responses to Toll-like receptor 2 stimulation. The biofilms of *P. gingivalis* grown in the presence of CSE showed lower pro-inflammatory capacity (involving cytokines TNF-alpha, IL-6, and IL-12) than control biofilms. Nicotine and *P. gingivalis* lipopolysaccharide (LPS) treatment of dendritic cells (DCs) modulates the immunopathogenesis of periodontal diseases. Nicotine combined with *P. gingivalis* or *P. gingivalis* LPS causes collagen degradation and bone resorption by tipping the balance between matrix metalloproteinases (MMPs) and tissue inhibitors of metalloproteinases (TIMPs). Smoking may compromise the immune response of patients with periodontitis against *P. gingivalis* antibodies, thereby increasing *P. gingivalis* infectivity.
Jiang et al. 2020. [2] Al Kawas et al. 2021. [13] Hanioka et al. 2019. [32] Imamura et al. 2015. [50]	Host cell invasion Conflicting results have been reported. Low concentrations of cigarette smoke condensate have been shown to increase the invasion of human gingival epithelial cells by *P. gingivalis*. The invasion of gingival epithelial cells was shown to increase near the wound area, and low concentrations of CSE and *P. gingivalis* can cause the inhibition of wound closure.

**Table 2:** Studies which have investigated the effect of smoking on peri-implantitis.

Studies	Methods	Findings
Leonhardt et al. (2003) [104]	A 5-year follow-up period of 26 implants with peri-implantitis following surgical and antimicrobial treatment.	Despite these therapies and a significant reduction in the presence of plaque and gingival bleeding, seven implants (26.9%) were lost. The authors considered smoking to be a negative risk factor for treatment success.
Roos-Jansåker et al. (2006) [106]	The effects of several potentially explanatory variables were analyzed in 218 patients treated with titanium implants for a period of 9–14 years after initial therapy.	Smoker patients were found to be more likely to develop peri-implantitis (univariate analysis: OR: 7.7, [98% CI: 2.5–14, *p* < 0.001); multivariate analysis: OR: 4.6 [98% CI: 1.1–19]) than nonsmokers (OR: 1.0 for both). On the patient level, smoking was associated with mucositis, bone level, and peri-implantitis (*p* = 0.02, < 0.001 and 0.002, respectively).
Sgolastra et al. (2015) [107]	A systematic review and meta-analysis study that assessed the role of smoking as a risk factor for peri-implantitis.	The implant-based meta-analysis revealed a higher and significant risk of peri-implantitis in smokers (RR: 2.1, 95% CI: 1.34–3.29, *p* = 0.001) compared with nonsmokers. The patient-based meta-analysis did not reveal any significant differences for risk of peri-implantitis in smokers (RR: 1.17, 95% CI: 0.78–1.75, *p* = 0.46).
Koldsland et al. (2009) [108]	This study assessed the outcome of dental implants inserted over a 16-year period.	The mean time from implant loading to the time of study was 8.4 years (range, 1.1 to 16.0 years). A total of 18 implants (4.8%) were lost out of 374 implants in 109 patients. The loss of oral implants was significantly associated with a history of smoking and periodontitis (*p* < 0.05).

**Table 3:** Studies which have utilized various periodontal surgical procedures in smoker and nonsmoker patients.

Study	Participant Group	Intervention	Results
Trombelli et al. (2003) [69]	19 smokers 12 nonsmokers	Periodontal parameters were assessed immediately before and 6 months following flap debridement surgery (FDS) surgery for Class I or II molar furcation defects.	Significantly more improvement was observed in nonsmokers than in smokers. v-CAL and h-CAL gain: Smokers: 1.07 ± 1.3 and 0.67 ± 1.0 mm Nonsmokers: 1.37 ± 1.1 and 1.37 ± 1.1 mm Class II furcation improved to Class I: Smokers: 27.6% Nonsmokers: 38.5% Class I furcation defects that completely healed: Smokers: 3.4% Nonsmokers: 27.8%
Boström et al. (1998) [70]	20 smokers 20 former smokers 17 nonsmokers	The 5-year outcome following periodontal surgery.	Periodontal probing depth (PPD) difference between follow-up and baseline (mean and SEM): Smokers: −1.0 (0.38) mm Former smokers: −1.6 (0.41) mm Nonsmokers: −1.2 (0.34) mm Periodontal bone height (PBH)% difference between follow-up and baseline (mean and SEM): Smokers: 1.7 (1.53) % Former smokers: 3.9 (1.67) % Nonsmokers: 7.7 (2.03) % Level of tumor necrosis factor alpha (TNF-a) in gingival crevicular fluid: Smokers: 71.7 (98.97) pg/mL Former smokers: 23.5 (23.19) pg/mL Nonsmokers: 15.7 (19.99) pg/mL
Scabbia et al. (2001) [71]	28 smokers 29 nonsmokers	Treatment outcome 6 months after flap debridement surgery for moderated to severe periodontitis patients.	Significantly more improvement was observed in nonsmokers than in smokers. Periodontal probing depth (PPD) reduction Smokers: 1.9 ± 0.7 Nonsmokers: 2.4 ± 0.9 Clinical attachment level CAL gain Smokers: 1.2 ± 0.7 Nonsmokers: 1.6 ± 0.7
Hellström et al. (2008) [72]	Control group (MWF): 17 smokers 13 nonsmokers Test group (M + MWF): 17 smokers 11 nonsmokers	The effects of minocycline microspheres (M) on periodontal probing depth reduction when used in combination with modified Widman flap (MWF) surgery in adults with moderate to severe chronic periodontitis.	Smokers in the test group had a significantly greater probing depth reduction (2.30 mm) than smokers in the control group (2.05 mm). PD reduction (mm) from baseline to weeks 13 and 25: Control group: Smokers: 2.17 ± 0.11 and 2.05 ± 0.09 Nonsmokers: 2.41 ± 0.16 and 2.37 ± 0.22 Test group: Smokers: 2.40 ± 0.11 and 2.30 ± 0.09 Nonsmokers: 2.55 ± 0.18 and 2.77 ± 0.24 Bleeding on probing (BoP) (%) reductions from baseline to weeks 13 and 25: Control group: Smokers: 64 ± 5 and 54 ± 4 Nonsmokers: 60 ± 7 and 59 ± 6 Test group: Smokers: 70 ± 5 and 66 ± 4 Nonsmokers: 53 ± 7 and 62 ± 6
Kaldahl et al. (1996) [120]	31 heavy smokers (HS) 15 light smokers (LS) 10 past smokers (PS) 18 nonsmokers (NS)	A total of 7 years of clinical outcomes of four treatment modalities (coronal scaling, root planing (RP), modified Widman surgery (MW), and flap with osseous resection surgery (OS)) for moderate to advanced periodontitis patients.	Following all phases of therapy, past smokers and nonsmokers consistently exhibited a significantly greater reduction in probing depth and clinical attachment gains. Mean reduction in probing depth and clinical attachment gain: HS: 1 mm, 0.2 mm LS: 0.8 mm, 0.4 mm PS: 2 mm, 0.2 mm NS: 1.9 mm, 1 mm
Ah et al. (1994) [121]	46 smokers 28 nonsmokers	A total of 6 years of clinical responses to nonsurgical and surgical periodontal therapy (coronal scaling, root planing (RP), modified Widman surgery (MW), and flap with osseous resection surgery (OS)) for moderate to advanced periodontitis patients.	CAL gain and recession level were less favorable in smokers than in nonsmokers. Mean clinical attachment gain and recession level reduction: Smokers: 0.5 mm and 0.8 mm Nonsmokers: 1 mm and 0.9 mm
Kim et al. (2007) [122]	19 smokers 22 nonsmokers	Assessed the effect of tooth-related and patient-related factors on the success of scaling and root planing (SRP) and access flap (AF) surgery in untreated and/or recurrent periodontitis patients.	RAL-V gain and PPD reduction were less favorable in current smokers. Backward multilevel linear regression analysis—dependent variable: RAL-V reduction 6 months after therapy: Smokers: −0.2875 (Estimate) 0.1106 (SE) Backward multilevel linear regression analysis—dependent variable: PPD reduction 6 months after therapy: Smokers: −0.3312 (Estimate) 0.1055 (SE) Multilevel linear regression analysis—dependent variable: RAL-V reduction 6 months after therapy: Smokers: −0.3758 (Estimate) 0.1334 (SE)
Orbak et al. (2003) [123]	25 smokers 25 nonsmokers	Gingival biopsies were taken from the pocket wall of chronic periodontitis patients and tested for CD4+, CD8+ lymphocyte, and CD4/ CD8 ratio values before treatment, after initial treatment, after curettage, and after flap operation.	Despite the use of different treatment methods, smokers had lower lymphocyte values than nonsmokers and a weaker local immune response. CD4+ and CD8+ lymphocyte values and CD4/CD8 ratio after curettage: Smokers: 27.00 ± 6.28, 13.69 ± 3.95, 2.02 ± 0.26 Nonsmokers: 33.55 ± 6.46, 17.36 ± 4.20, 1.97 ± 0.36 CD4+ and CD8+ lymphocyte values and CD4/CD8 ratio after flap surgery: Smokers: 28.85 ± 4.83, 15.85 ± 2.76, 1.83 ± 0.20 Nonsmokers: 33.73 ± 6.61, 18.36 ± 3.11, 1.84 ± 8.73

SEM: standard error of means; CAL: clinical attachment level; v-CAL: vertical clinical attachment level; h-CAL: horizontal clinical attachment level; PPD: probing pocket depth; RAL-V: vertical relative attachment level.

## Data Availability

All data supporting the findings of this study will be available from the corresponding author [M.M.] upon request.
